# Omicron variant: a booster depending on infection histories

**DOI:** 10.1038/s41392-022-01279-2

**Published:** 2023-01-04

**Authors:** Jiayu Wang, Tianxia Lan, Yuquan Wei, Yoshimasa Tanaka

**Affiliations:** 1 State Key Laboratory of Biotherapy, Cancer Center, West China Hospital, Sichuan University and Collaborative Innovation Center for Biotherapy, 610041 Chengdu, China; 2 Center for Medical Innovation, Nagasaki University, 1-7-1 Sakamoto, Nagasaki, 852-8588 Japan

**Keywords:** Infectious diseases, Infectious diseases, Vaccines

Recently, Reynold et al. published a study in *Science*^1^ showing that immune imprinting patterns might differentially influence immune responses against variants of concern (VOCs) of severe acute respiratory syndrome coronavirus 2 (SARS-CoV-2). They also demonstrated that infection with B.1.1.529 (Omicron variant) of SARS-CoV-2 confers only partial and transient protection against itself, resulting in frequent reinfections with Omicron in short time intervals.

**Fig. 1:**
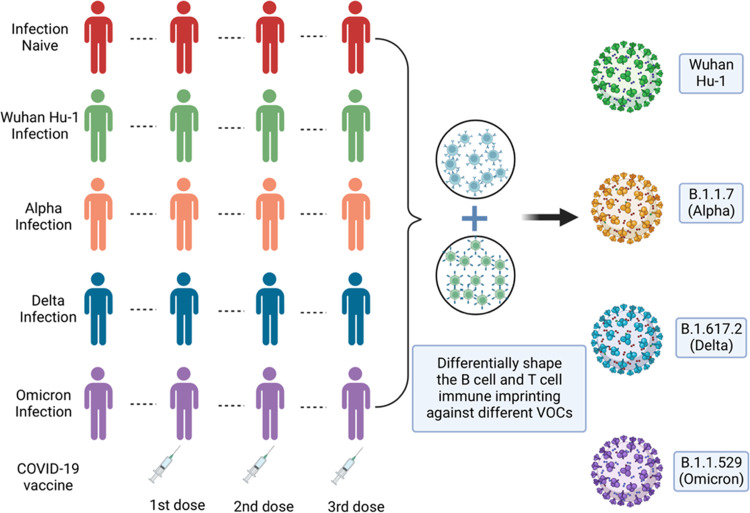
SARS-CoV-2 infection history and Omicron infection alter and define T- or B-cell immunity in triple-vaccinated HCWs. Triple-vaccinated HCWs with different SARS-CoV-2 infection histories were examined for T- and B-cell immunity toward VOCs, including the Omicron. Different combinations of SARS-CoV-2 infection and vaccination led to distinct, imprinted patterns of hybrid immunity. Although hybrid priming by infection and vaccination was generally expected to enhance protective immunity, a combination of Wuhan Hu-1 or Alpha infection and three doses of vaccines had a negative effect on protective immunity against the Omicron infection. The precise molecular mechanism underlying the phenomenon, “hybrid immune damping”, should be elucidated for the future development of effective vaccines. This figure was generated based on ref. ^1^

In late 2021, the Omicron variant emerged and rapidly replaced the previously dominant variants. With 36 mutations in the spike domain, which consists of the S1 and S2 spike subunits, Omicron manifests higher transmissibility and immune evasion than earlier variants. Although vaccination and SARS-CoV-2 exposure produce different levels of protection against infection and reinfection with a variety of variants,^2,3^ it is still unclear how distinctive histories of infections differentially shape immune responses against Omicron. Furthermore, it is unknown whether Omicron infection can act as a natural booster of immunity against SARS-CoV-2 VOCs. To address these questions, Reynold et al. analyzed adaptive immune responses in BNT162b2 mRNA-vaccinated London healthcare workers (HCWs) with different histories of SARS-CoV-2 infections (Fig. 1).

Reynold et al. first demonstrated that antibody responses and memory B cell (MBC) counts were lower in HCWs who had received three doses of mRNA vaccine when they were infected with Omicron than those infected with Wuhan Hu-1 or other VOCs. These results showed that previous infection(s) and heterologous antigen exposure could alter and define immune responses to future infection(s) with VOCs, which agrees with the results from previous studies.^4^ In addition, HCWs who were infected with the ancestral Wuhan Hu-1 had lower antibody responses against B.1.351 (β variant), P.1 (γ variant), and Omicron variant than infection-naïve HCWs. Interestingly, HCWs who had been infected with both Wuhan Hu-1 and B.1.617.2 (δ variant) showed enhanced neutralizing antibody (nAb) responses against Wuhan Hu-1 and δ variants. However, serum nAb responses against the Omicron variant were markedly diminished, when compared to the other VOCs, regardless of the history of SARS-CoV-2 infections. Moreover, MBC frequencies against the Omicron variant 2–3 weeks after the third vaccination and 20–21 weeks after the second vaccination were significantly lower than those against the ancestral Wuhan Hu-1 and δ variants, regardless of the infection history.

In addition, responses of T cells against Omicron were significantly weaker than those against Wuhan Hu-1 and δ variants. Based on these findings, patterns of T-cell responses against a mapped epitope pool (MEP) derived from Omicron spike subunits S1 and S2, and a matched pool from Wuhan Hu-1 were compared to explore how Omicron spike mutations influence T-cell recognition. It is worth noting that T-cell responses against the Omicron MEP were lower than those against the matched Wuhan Hu-1 pool, regardless of the infection history, suggesting that the cross-recognition by T cells of the Omicron spike epitopes was reduced in triple-vaccinated HCWs.

Omicron spike mutations led to a loss of HLA-DR4-restricted T-cell epitopes at four specific sites and a gain of nascent HLA-DR4 T-cell epitopes at four additional sites. To analyze the gain and loss of T-cell epitopes associated with Omicron spike mutations, the ancestral Wuhan Hu-1 or Omicron sequence-specific peptide pool was used to immunize HLA-DRB*04:01 transgenic mice. Intriguingly, priming T-cell responses with one pool led to reduced responses to the other, suggesting a causative mechanism underlying “hybrid immune damping”, wherein protection against future infection might be altered by incidental immune imprinting patterns.

Reynold et al. further analyzed B-cell immunity following Omicron infection and demonstrated that triple-vaccinated HCWs who were infected with Omicron as the first infection showed increased MBC frequency and antibody responses against all VOCs, including Omicron. However, cross-reactive antibody responses in triple-vaccinated HCWs who were infected initially with the ancestral Wuhan Hu-1 and later with Omicron were not significantly induced, compared to those in uninfected HCWs. Moreover, infection-naïve HCWs showed only marginal antibody responses against Omicron.

Reynold et al. next determined T-cell responses against Omicron infection in triple-vaccinated HCWs. Surprisingly, Omicron infection failed to induce significant T-cell responses against itself, regardless of the infection history. The observation that T-cell responses against Omicron were impaired in triple-vaccinated HCWs who were infected initially with the ancestral Wuhan Hu-1 and later with Omicron is consistent with reduced antibody responses against Omicron, suggesting that Omicron infection is not a powerful natural booster against itself, especially for HCWs who were infected during the Wuhan Hu-1 wave. On the other hand, infection with Omicron was found to enhance cross-reactive T-cell immune recognition against spike MEPs of the Wuhan Hu-1 and δ variant.

Finally, by analyzing B- and T-cell responses in the longitudinal HCW cohort, Reynold et al. demonstrated that hybrid immunity (single vaccination plus prior infection) could lead to a significant increase in antibody responses against Omicron. Besides, for HCWs who experienced infection with Wuhan Hu-1 or α variant, antibody responses against Omicron were significantly increased 2–3 weeks after the second vaccination and then declined after 20–21 weeks. Importantly, triple-vaccinated HCWs who experienced the ancestral Wuhan Hu-1 infection showed no increase in antibody-binding responses against Omicron. In short, immune imprinting mediated by Wuhan Hu-1 significantly diminished the antibody responses against Omicron in HCWs who were infected with Omicron.

In summary, this study demonstrated that various infection and vaccination histories, so-called immune imprinting, might alter adaptive immune responses against the Omicron variant. Accordingly, immune responses against Omicron were not effectively induced by previous infections with Wuhan Hu-1 or other SARS-CoV-2 VOCs in triple-vaccinated HCWs. In addition, Omicron infection failed to serve as a natural immune booster against itself. On the other hand, antibody responses against Omicron were induced in several cohorts of HCWs with specific histories of infection and vaccination. These findings may provide novel insights into the development of effective anti-Omicron strategies.
